# Policy Brief – Survivorship is cancer survivorship the poor cousin of cancer control within the EBCP?

**DOI:** 10.1186/s13690-024-01371-9

**Published:** 2024-08-29

**Authors:** Régine Kiasuwa Mbengi, Gabrielle Schittecatte, Sofie Theys

**Affiliations:** Belgian Cancer Centre, Sciensano, Brussels, Belgium

**Keywords:** Cancer survivorship, Survivorship care, Quality of life, Quality of care

## Abstract

**Background:**

There is an increasing number of cancer survivors, including children, adolescents, young adults, individuals of working age, and the elderly, within the Belgian and European population. Yet, survivorship care and rehabilitation are often treated as an afterthought in care organisation. This not only directly affects the quality of life of survivors and carers, but also puts the sustainability of the healthcare and social security systems at risk.

**Methods:**

We analysed the ongoing Europe Beating Cancer Plan (EBCP) to identify the actions supporting survivorship (care) developments, then compared their weight in the EBCP to the other domains of cancer control. Following this analysis, and comparison with related ongoing projects, and current infrastructure in Belgium, several unmet needs were identified.

**Conclusion:**

To better address these unmet needs, we recommend that Belgium incorporates survivorship care and long-term follow-up in clinical guidelines and care pathways, and considers including indicators related to cancer survivorship in the planning and design of quality insurance schemes, including certification of comprehensive cancer centres. Furthermore, we suggest further investment and support for research and knowledge exchange in the field of survivorship.

## Introduction

The prevalence of cancer survivors is growing every year (around 3% per year), and is now estimated at over 12 million survivors in Europe, with about 300.000 childhood cancer survivors [1]. The five year survival rates for all cancers combined is 71% for the 2014–18 period in Belgium [2].

Cancer survivors experience a wide range of problems related to physical and mental functioning, including symptoms such as fatigue and pain, sexual problems, issues related to body image, distress, fear of recurrence, in addition to cognitive, social and physical functioning [3].

Improvements in prevention, diagnostics and treatment are key elements for cancer control, leading to increases in survival. However, relative to the medical and technical developments, there is less focus placed on adequately addressing the plethora of challenges associated with cancer survivorship.

In spite of relative recent attention attributed to survivorship, the work that remains is colossal [4, 5]. It is urgent to address these challenges to ensure high quality long-term care and quality of life for cancer survivors.

## Methodology

The issue overview, gaps, and recommendations were identified in a participative manner with Belgian Europe’s Beating Cancer Plan (EBCP) Mirror Group on Survivorship. The group consists of more than 50 members, with representatives from patient organizations, NGOs, hospitals, professional organizations, registries and universities.

Building off evidence-based recommendations on survivorship from the Joint Action CanCon [6], the Belgian EBCP Mirror Group on Survivorship [7] developed concrete recommendations to improve survivorship care in Belgium. They did so by reviewing the 42 actions of the EBCP and assessing these actions for opportunities to address cancer survivorship issues. They then compared the actions in EBCP and the evidence based recommendations from CanCon with current open calls and/or ongoing projects at the EU-level in which cancer survivorship is addressed. Synergies with the Belgian context were then identified and the extent to which the EBCP projects could benefit to the Belgian Handbook for Supportive Care were assessed (Table 1). The main objective of this work was to identify the challenges hindering the rehabilitation and the quality of life of cancer survivors.

**Table 1:** The selected list of EBCP actions and their potential impact for survivorship (care) and related infrastructure in Belgium, and the gaps between the two

Action	The flagship, initiative or action^a^	Possible impact on survivorship care^b^	Planned or ongoing projects including survivorship^c^	Existing infrastructure/activities in BE^d^	Persistent Gaps in BE^e^
1	‘Knowledge Centre on Cancer’	Indicators to monitor the QoL of survivors, the quality of survivorship care, access to rehabilitation, return-to-work	Currently no data related to cancer survival, survivorship or quality of life of survivors is included in the ECIR^f^	No infrastructure	No information system in Belgium for survivorship
13	Occupational Safety and Health Strategic Framework 2021–2027	Inclusion of binding legal basis for the protection of workers with cancer and job maintenance	The EU strategic framework on health and safety at work 2021-2027^g^	The plan for the reintegration of patients in (long-term) sick leave (but is not cancer specific)	The BE plan for work reintegration is not adapted to cancer survivors and people report difficulties finding the right and updated information
23.1	National Comprehensive Cancer Centre(s)	Inclusion of prerogatives for CCCs in cancer survivorship care and rehabilitation	One task of the JA CraNE addresses survivorship/return-to-work pathway^h^	Minimal requirements to be recognized ‘oncological care program’ (OCP) Some OCPs recently decided to create ‘survivorship clinics’ in their institutions	No requirements related to (long-term) survivorship care in the legal basis of Oncological Care Programmes
23.2	New cancer Reference Networks	Share expertise on survivorship	One work package of the JA Network of Expertise is dedicated to survivorship	Some OCPs recently decided to create ‘survivorship clinics’ in their institutions.	
25	European Initiative to Understand Cancer (UNCAN.eu)	Ensure a place for survivorship care and quality of life of survivors on the national and EU research agendas	UNCAN.eu put survivorship on the EU strategic agenda for cancer research^i^	While the BSMO has a Survivorship Taskforce, there is no national or regional coordinated initiatives for cancer survivorship research in Belgium	Cancer survivorship research in Belgium needs to be put at the agenda of funders
26	Inter-specialty trainingsprogramm	Ensure the training of professionals in survivorship care and rehabilitation and facilitate expertise and knowledge sharing	Nothing started	No valorised training for cancer survivorship in BE	Specializations in oncology do not require work with oncological patients or a shift in late effect clinic, however theory is taught in classes
31.1	Partnership on Precision Medicine	Needed to anticipate the rehabilitation needs	Nothing started	Many personalized medicine initiatives in BE arose but none of them address cancer survivorship	The inclusion of survivorship in personalized pathways
34.1	Cancer Survivor Smart-Card	Improvement of survivorship needs and care registration	Ongoing	There is a pilot for the introduction of the survivorship passport	No electronic records are available to register and monitor survivor’s physical or psychosocial information
34.2	European Cancer Patient Digital Centre				
35	Fair access to financial services	Improve long-term reintegration and QoL	Ongoing	The right to be forgotten is implemented in Belgium since 2019	The Royal Decree of 26th May 2019 implementing the right to be forgotten
36.1	Return to work	Practical solutions to support RTW	Ongoing	Several studies have been performed in BE to understand the issues related to RTW after cancer	More efforts should be dedicated to the development of interventions and cost-effectiveness studies
41	EU Network of Youth Cancer Survivors	Improve transnational cooperation and efforts to improve QoL of CAYAs	Several calls are open Different projects of the PanCare society	A project for a convention to better support and organize psychosocial care for children is under development	An organized transition from childhood to adulthood post cancer care is still lacking, although some recent initiatives are supported

^a^This column describes the flagship or action of the EBCP related to survivorship

^b^This column describes the possible impact the action has on survivorship care

^c^This column describes the any ongoing projects related to the action and survivorship

^d^This column details any existing projects related to the action taking place in Belgium

^e^This column details the gaps that still exist in Belgium related to the action, given the ongoing projects and current infrastructure in Belgium. These gaps form the basis of the below recommendations

^f^EU Joint Research Center. European Cancer Inequalities Registry data tool: https://cancer-inequalities.jrc.ec.europa.eu/data-tool-by-country?ind=ALLMORT&ft=TOTAL

^g^European Commission. 28 June 2021. Communication from the Commission to the European Parliament, the Council, the European Economic and Social Committee and the Committee of the Region. COM(2021) 323 final. EU strategic framework on health and safety at work 2021–2027 Occupational safety and health in a changing world of work. https://eur-lex.europa.eu/legal-content/EN/TXT/PDF/?uri=CELEX:52021DC0323&from=EN

^h^Joint Action on European Network of Comprehensive Cancer Centres. WP8 Equitable Access to high-quality care & research network in the context of CCCs. https://crane4health.eu/wp8-equitable-access-to-high-quality-care-and-research-networks-in-the-context-of-cccs/

^i^Solary E. et al. UNCAN.eu, a European Initiative to Understand CANcer. Cancer Discovery November 2022. https://uncan.eu/wp-content/uploads/2023/01/UNCAN_eu-a-European-Initiative_Eric-Solary-et-al_IN-FOCUS-2022.pdf

### Issue overview

Survivorship has often taken a back seat to other elements in the patient pathway. Treatment toxicities, physiological and psychosocial (late) effects, overconsumption of drugs and drug interactions, management of comorbidities, etc., should be at the heart of survivorship initiatives in Belgium, but also at the EU level. Survivorship remains important for the entire Belgian and EU population; from youth, to people aged over 65 years. The latter remains particularly important as while Europe is an aging continent, most Member States do not consider survivorship care as a necessity in public health care systems.

In 2017, under the framework of the Joint Action CanCon, EU experts worked together to develop evidence-based recommendations to improve the integration of cancer survivorship and rehabilitation in national cancer control programs [8]. In addition to these recommendations, the Mirror Group’s review of the EBCP actions, and discussions of opportunities for survivorship in Belgium, resulted in four priority proposals:

I. Creation of a national cancer survivorship portal to improve the ‘information and communication’ among professionals and patients
II. The development of a generic cancer survivorship care pathway (SCP) and the setup of a recovery convention, ensuring equal and high-quality survivorship care for all cancer patients
III. A pilot of the SCP and research efforts for its monitoring and evaluation
IV. Development of local networks of oncological aftercare

The submission of these recommendations for action to the Ministry of Public Health and the underlying discussions led to the decision to start with the development of a Handbook for Cancer Supportive Care [9].

In the following parts of this policy brief we will (1) examine the extent to which the activities in Belgium and in the EBCP address the areas for development, and (2) present recommendations for persistent gaps that are not currently addressed.

### Survivorship in the EBCP: results from a gap analysis

We screened the 42 actions of the EBCP on their opportunity and relevance to address cancer survivorship. Three out of the 42 actions of the EBCP directly address survivorship. Chapter 6 of the EBCP, ‘Improving the Quality of Life for Cancer Patients, Survivors, and Carers’, includes the most initiatives related to cancer survivorship. The first of these actions is the flagship ‘Better life for cancer patients’, which focuses on the development of the Cancer Survivor Smart-Card (34.1) and the creation of a framework for data storage and exchange (34.2). The second is action 35, which foresees the development of a code of conduct for access to financial services, which can be regarded as a step back compared to the Right to be forgotten. The third is action 36, which concerns the legal frameworks of the work conditions for cancer survivors and their social security status. It should also be noted that the terms ‘survivors’ and ‘survivorship’ also appear in chapters 5.1 on ‘delivering higher-quality care’ (for the creation of an EU network of expertise), 5.2 on ‘ensuring a high-quality workforce’ (for the training of the health workforce), and 8 on ‘putting childhood cancer under the spotlight’.

Twelve other actions were identified that could potentially have an impact on cancer survivorship. This offers considerable room for manoeuvre, and to highlight the topic of survivorship in future projects and work programmes.

The following table presents the aforementioned actions, their potential impact for survivorship, the possible impact of the action on survivorship care, followed by a presentation of the existing infrastructure in Belgium, and the gaps between the objective of the action and the infrastructure in Belgium. This final column details the gaps and is the basis of the policy recommendations presented in the subsequent section.

### Policy recommendations

The EBCP funded projects and programs are still ongoing and the overview provided in this policy brief is not comprehensive. Cancer survivors are not forgotten in the EBCP and several chapters include them in some extent. In Belgium, several initiatives related to survivorship have been identified. However, unmet needs among cancer survivors are still numerous [10]. In this section we detail our recommended actions to overcome the challenges associated with these unmet needs.

First, through our analysis, it is clear that high quality survivorship care and its organization are side-lined in chapter 5 (high standards in cancer care). This creates unnecessary partitioning between survivorship care and the diagnostic and treatment phases. Even information systems and monitoring between diagnostics and treatment, and survivorship seems to be the purpose of an distinct initiative (flagship 8). This does not lend to the integration of survivorship care in future care organization developments. This sends a wrong signal to healthcare authorities and professionals. Given that more cancer patients will survive in the future, Belgium and EU countries should implement best practices, and include survivorship care in their clinical guidelines as well as in their care pathways.

Second, at both Belgium and EU levels, the scarce attention given to survivorship in the projects addressing the organization of cancer care, represents missed opportunities to integrate survivorship care in standardized care pathways. This is of tremendous importance as the criteria and indicators being developed, such as those for the certification of comprehensive centres, will exclude survivorship care and rehabilitation from their scope. Health authorities and administrations must pay attention to the provision of indicators related to cancer survivorship when planning and designing quality insurance schemes, including certification of centres.

Third, although recently cancer patients are benefitting from better treatments and care organization, the organization of supportive care is lacking, for various reasons [10]. There is a need to ensure that cancer patients and survivors also benefit from innovations, just as patients under treatment do. In that vein, support is required to foster more research and knowledge exchange, to confront the lack of evidence in many areas of survivorship.

In conclusion, cancer survivorship care remains an afterthought in care organisation, as if it were an add-on. Given the increase of cancer survivors, their unmet needs may put the sustainability of healthcare and social security systems at risk, and more importantly, their wellbeing and social inclusion. The underlying glaring inequity between survivorship and other cancer care domains is not acceptable and decision-makers should concentrate the efforts in subsequent work programs to get back on track to guarantee quality of life and access to high-quality survivorship care for the 12 million European cancer survivors and their relatives.

## Data Availability

Not applicable.

## Abbreviations

EU: European Union
EBCP: Europe’s beating cancer plan
QoL: Quality of Life
SCP: Survivorship care pathway
JA CanCon: Joint action on cancer control
